# Down-regulation of miR-675-5p contributes to tumor progression and development by targeting pro-tumorigenic GPR55 in non-small cell lung cancer

**DOI:** 10.1186/s12943-015-0342-0

**Published:** 2015-04-01

**Authors:** Dan He, Jun Wang, Chunfang Zhang, Bin Shan, Xiyun Deng, Bin Li, Yanwu Zhou, Wei Chen, Jidong Hong, Yang Gao, Zhuchu Chen, Chaojun Duan

**Affiliations:** Key Laboratory of Cancer Proteomics of Chinese Ministry of Health, Institute of Medical Sciences, Xiangya Hospital, Central South University, Xiangya Road 87th, Changsha, 410008 Hunan PR China; College of Medical Sciences, Washington State University Spokane, 412 E.Spokane Falls Boulevard, Spokane, WA 99202 USA; Faculty of Basic Medical Sciences, College of Medicine, Hunan Normal University, Changsha, 410013 PR China

**Keywords:** miR-675-5p, Progression, NSCLC, GPR55

## Abstract

**Background:**

microRNAs are small noncoding RNAs that modulate a variety of cellular processes by regulating multiple targets, which can promote or inhibit the development of malignant behaviors. Accumulating evidence suggests that miR-675-5p plays important roles in human carcinogenesis. However, its precise biological role remains largely elusive. This study examined the role of miR-675-5p in non- small cell lung cancer (NSCLC).

**Methods:**

The expression of miR-675-5p was analyzed by real-time quantitative PCR (qRT-PCR). The effect of miR-675-5p on proliferation was evaluated through MTT and colony formation assays, and cell migration and invasion were evaluated through transwell assays. The expression of target proteins and downstream molecules was analyzed by western blotting and immunohistochemical staining. The luciferase reporter assay was used to assess the target genes of miR-675-5p in non-small cell lung cancer cells.

**Results:**

The expression levels of miR-675-5p in NSCLC tissues were significantly reduced compared to those in adjacent non-cancerous tissues (P < 0.001). The expression of miR-675-5p in patients with non-small cell lung cancer had a negative correlation with lymph node metastasis (P < 0.01) and TNM stage (P < 0.05). Down-regulation of miR-675-5p promoted cell growth, proliferation, colony formation, invasion and migration, and promoted the tumorigenicity graft growth of nude mice in vivo (P < 0.01); whereas up-regulation of miR-675-5p had the contrary effects. The luciferase reporter assay showed that GPR55 was a direct target gene of miR-675-5p. Overexpression of miR-675-5p can lead to the down-regulation of GPR55 and its signaling pathway, whereas the effect can be reversed by down-regulation of miR-675-5p expression.

**Conclusions:**

miR-675-5p functions as a novel tumor suppressor in NSCLC and the anti-oncogenic activity may involve its inhibition of the target gene GPR55. These findings suggest the possibility for miR-675-5p as a therapeutic target in NSCLC.

**Electronic supplementary material:**

The online version of this article (doi:10.1186/s12943-015-0342-0) contains supplementary material, which is available to authorized users.

## Background

Lung cancer is a malignant tumor with the highest morbidity and mortality in the world, which is a serious threat to human health and life security [1]. Non-small cell lung cancer (NSCLC) accounts for about 80% of all lung cancer cases, including adenocarcinoma and squamous cell carcinoma [2]. Early lung cancer patients usually lack obvious symptoms for early diagnosis, and mostly are late stage lung cancer when diagnosed. In recent years, although predominantly surgical comprehensive treatment has achieved great progress, the 5-year survival rate is still less than 15% [3]. Therefore, investigations of the molecular mechanisms underlying progression and metastasis of NSCLC can help develop novel prognostic biomarkers and therapeutic targets for the malignancy, and thus are clinically important. A large number of studies have shown that abnormal expression of microRNAs (miRNAs) is closely related to the development of NSCLC [4-8]. Therefore, as a kind of new molecular target, miRNAs have attracted tremendous attention in oncology and other biomedical research fields.

miRNAs are a class of small noncoding RNAs (19 ~ 24 nucleotide) that regulate the expression of target genes through binding to the 3′- untranslated region (3′ UTR) of target genes, resulting in translational repression or mRNA degradation. miRNA not only can function as a tumor suppressor gene through down-regulation of oncogene activation, but also as a oncogene through down-regulation of tumor suppressor gene activation [9,10]. Dysregulation of miRNAs may lead to many pathological processes that are very important in the development of cancerous alterations, such as cellular tumorigenesis, differentiation, proliferation, apoptosis, mobility, and invasion [11-14]. Thus, understanding of the underlying molecular mechanisms of miRNA dysregulation in malignant tumors is critical to intervention of lung cancer.

Recent studies have shown that miR-675 expression was up-regulated in several cancer types, such as glioma [15], gastric cancer [16,17], colorectal cancer [18] and hepatocellular cancer [19]. Another study found low expression of miR-675 in adrenal cortical carcinoma and metastatic prostate cancer cells [20,21], implying that miR-675 may play different roles depending on the tumor type. In this study, we aimed to evaluate the possible roles and related target genes of miR-675-5p in tumorigenesis of NSCLC. We found that the expression level of miR-675-5p was significantly lower in NSCLC tissues than in the corresponding normal lung tissues, and inversely associated with advanced stage and lymph node metastasis of NSCLC. Furthermore, enforced miR-675-5p expression inhibited lung cancer cell growth, proliferation, clone formation, migration and invasion in vitro, and tumorigenicity in vivo. In addition, THE orphan G protein-coupled receptor 55 (GPR55) was identified as a functional target of miR-675-5p. Therefore, down-regulation of miR-675-5p suppresses lung cancer progression and metastasis through regulation of GPR55.

## Results

Expression of miR-675-5p is inversely associated with advanced stage and lymph node metastasis of NSCLC.

**Figure 1:**
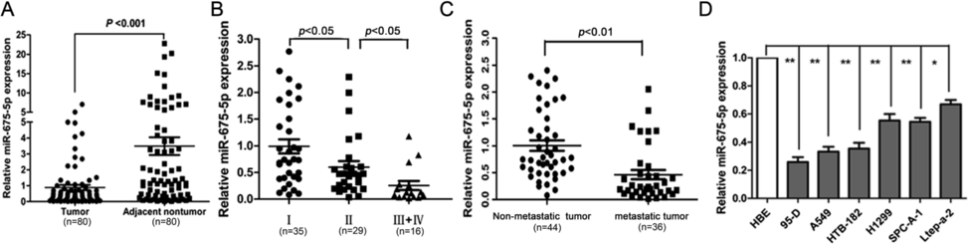
**Down-regulation of miR-675-5p is inversely associated with advanced stage and lymph node metastasis of NSCLC. (A)** miR-675-5p expression level was significantly lower in NSCLC tissues than in their matched normal tissues. **(B)** Low-level expression of miR-675-5p was associated with high tumor stage of NSCLC (P < 0.05). **(C)** Low-level expression of miR-675-5p was related with lymph node metastasis of NSCLC (P < 0.05). (**D)** miR-675-5p expression in NSCLC cell lines and normal human bronchial epithelial cell line (HBE). Expression levels of miR-675-5p were determined by qRT-PCR and normalized against an endogenous control (U6 RNA). Data were represented as the mean ± SEM of three independent experiments. *P < 0.05, **P < 0.01.

We noticed that miR-675-5p was underexpressed in NSCLC by using microarray (our unpublished data). To confirm reduced expression of miR-675-5p in NSCLC, we evaluated the expression of miR-675-5p in 80 pairs of frozen NSCLC tissues and the corresponding normal lung tissues using quantitative reverse transcriptase PCR (qRT-PCR). The RNA levels of miR-675-5p in NSCLC tissues were less than 30% of that in the matching normal tissues (Figure 1A). miR-675-5p expression was significantly inversely associated with metastasis and Classification of Malignant Tumours (TNM) classification of the patients (Table 1, P < 0.005). Furthermore, the expression level of miR-675-5p in tumor tissues decreased statistically with increasing stage of NSCLC (P < 0.05) (Figure 1B). In addition, miR-675-5p expression was significantly lower in NSCLC that displayed lymph node metastasis than in NSCLC that did not (P = 0.0055). Therefore, the low miR-675-5p expression was closely related to the progression and metastasis of NSCLC. We also measured miR-675-5p expression in six NSCLC cell lines (95-D, A549, HTB-182, NCI-H1299, SPCA-1, Ltep-a-2) and a normal human bronchial epithelial cell line (HBE). The relative expression levels for miR-675-5p in these six NSCLC cell lines were 0.224, 0.343, 0.378, 0.562, 0.541, and 0.673, respectively, as compared with that of HBE cells, respectively (Figure 1D).

**Table 1:** **miR-675-5p expression and clinicopathological features in non-small cell lung cancer(NSCLC) patients**

**Variable**	**miR-675-5p expression**			
	**Cases**	**Low**	**High**	***P-value*** ^***a***^
Age(years)^b^				
<57	58	43 (74%)	15 (26%)	0.8998
≥57	22	16 (73%)	6 (27%)	
Gender				
Male	33	24 (73%)	9 (27%)	0.8750
Female	47	35 (74%)	12 (26%)	
Smoking history (years)^b^				
<10	36	24 (67%)	12 (33%)	0.2094
≥10	44	35 (80%)	9 (20%)	
Pathological type				
adenocarcinoma	38	30 (79%)	8 (21%)	0.3391
squamous carcinoma	42	29 (69%)	13 (71%)	
Tumor differentiation				
I + II	46	37 (80%)	9 (20%)	0.0787
III + IV	34	22 (65%)	12 (35%)	
TNM Classification				
I	35	19 (54%)	16 (46%)	<0.005
II	27	24 (89%)	3 (11%)	
III + IV	18	16 (89%)	2 (11%)	
Metastasis				
No	44	27 (61%)	17 (39%)	0.0055
Yes	36	32 (89%)	4 (11%)	

Abbreviations: TNM, tumor-node-metastasis; ^a^×^2^test. ^b^Mean age.

Overexpression of miR-675-5p inhibits proliferation, colony formation, migration, and invasion of NSCLC cells.

**Figure 2:**
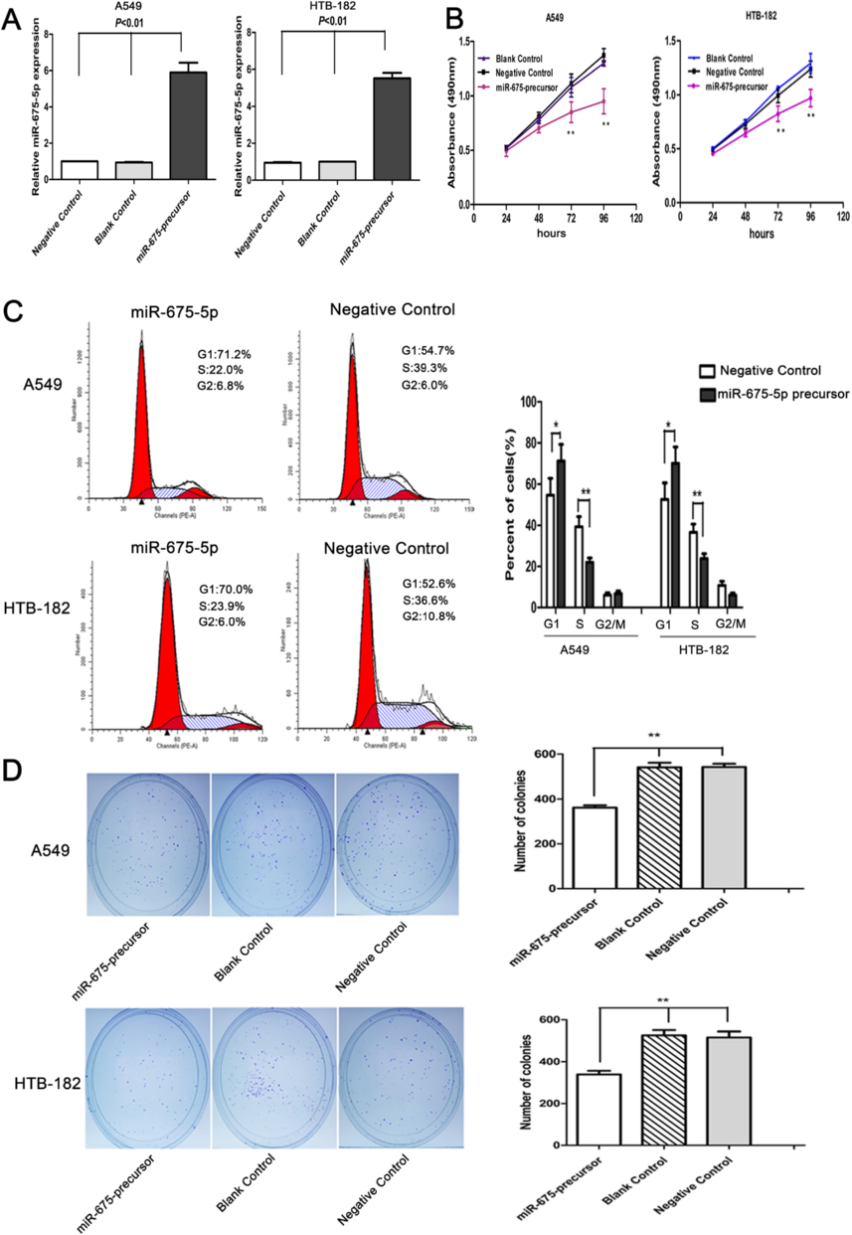
**Overexpression of miR-675-5p inhibited proliferation and colony formation of NSCLC cells. (A)** the level of miR-675-5p in A549 and HTB-182 cells was significantly up-regulated after transfection with miR-675-precursor. **(B)** miR-675-5p reduced cell proliferation in NSCLC cells. Cell proliferation was determined using MTT assays. **(C)** miR-675-5p induced cell cycle arrest at the G1/S phase. **(D)** miR-675-5p suppressed colony formation compared with controls. The number of colonies were calculated and depicted by the ban graph. Data were represented as the mean ± SEM of three independent experiments. Negative control: pGCsil-GFP Vector. *P < 0.05, **P < 0.01.

**Figure 3:**
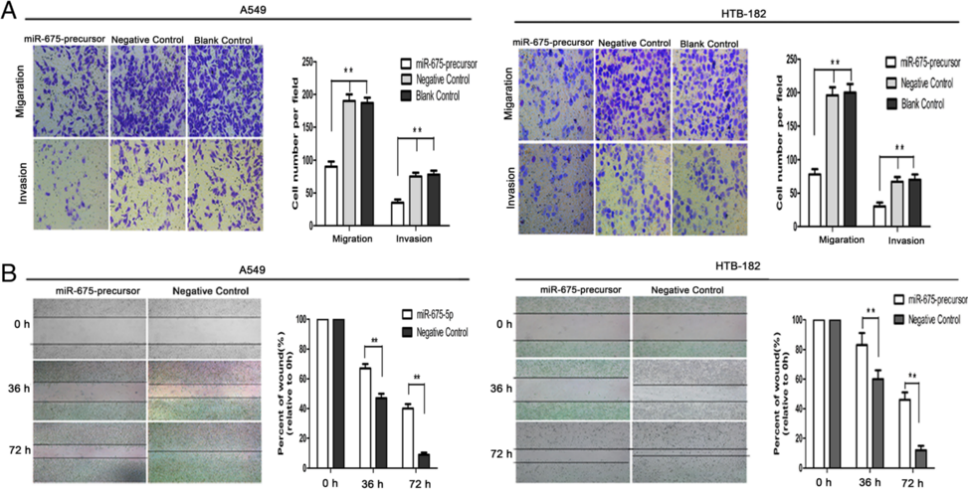
**The effect of miR-675-5p on in vitro migration and invasiveness of NSCLC cells. (A)** The number of migrating or invading cells in the miR-675-precursor group was significantly decreased compared with the control. **(B)** The wound healing rate in cells transfected with miR-675-precursor was significantly decreased. Data were represented as the mean ± SEM of three independent experiments. Negative control: pGCsil-GFP Vector. *P < 0.05, **P < 0.01.

As a low level of miR-675-5p expression in NSCLC is a common molecular incident and is correlated with progression of the disease, we hypothesize that overexpression of miR-675-5p in NSCLC can exert inhibitory effects on cell growth and proliferation. To validate the hypothesis, we transfected LV-miR-675-precursor or scrambled sequence (negative control) into A549 and HTB-182 NSCLC cells that had low basal levels of miR-675-5p in NSCLC cell lines (Fig1D). Interestingly, methylthiazol tetrazolium assay (MTT) showed that forced expression of miR-675-5p impaired the growth rate of the NSCLC cells (Figure 2A). Similarly, colony formation assays showed that cell proliferation in both A549 cells and HTB-182 cells were significantly repressed by forced expression of miR-675-5p (Figure 2C). To explore the possible mechanism underlying the inhibitory effect on cell growth by overexpression of miR-675-5p, cell cycle analysis was performed (Figure 2B). Upon up-regulation of miR-675-5p, the percentage of A549 and HTB-182 cells in G0/G1 phase increased from 54.7% ± 8.1% in controls to 71.2% ± 8.5% and from 52.6% ± 8.0% in controls to 70.0% ± 8.6%, respectively (P < 0.01), indicating that overexpression of miR-675-5p resulted in G1 phase arrest in NSCLC cells. Annexin V fluorescein isothiocyanate (V-FITC) apoptotic assay showed that there was no significant difference of apoptotic rate between the cells transfected with LV-miR-675-precursor and control cells (P > 0.05) (data not shown). Therefore, overexpression of miR-675-5p might reduce cell proliferation of NSCLC mainly through G1 phase arrest. Furthermore, miR-675-5p overexpression suppressed the migratory and invasive abilities of the NSCLC cells as determined by transwell assay (Figure 3A). Similarly, mobility of A549 and HTB-182 cells in wound healing assays was significantly decreased after transfection of miR-675-5p (Figure 3B). Taken together, miR-675-5p might have tumor-suppressor function.

Overexpression of miR-675-5p inhibits tumor growth of NSCLC cells in vivo.

**Figure 4:**
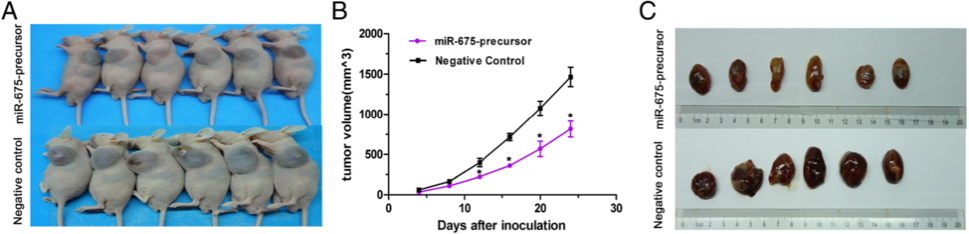
**Overexpression of miR-675-5p inhibits NSCLC in vivo. (A)** Tumor volumes of subcutaneous implantation models of NSCLC are shown. **(B)** Tumor growth curves of subcutaneous implantation models of NSCLC. **(C)** Tumor volumes in the orthotopic implantation models at week 4 are shown. Data were represented as the mean ± SEM of three independent experiments. Negative control: pGCsil-GFP Vector *P < 0.05.

Given that miR-675-5p impaired the proliferation, migration and invasion of NSCLC cells in vitro, we examined whether miR-675-5p could affect tumorigenicity in vivo. A549 cells stably expressing miR-675-5p and negative control vector were injected subcutaneously into nude mice. Palpable tumors formed within 1 week. Tumor volume was measured each week, and mice were sacrificed 4 weeks after tumor cell implantation (Figure 4A). The size of NSCLC tumors in these two groups was calculated and compared. The average tumor volume of A549 cells stably transfected with miR-675-5p was 1.23 ± 0.096 cm^3^, which was significantly smaller than tumors in the negative control group (1.86 ± 0.132 cm^3^) (Figure 4B). The tumor growth-curve of tumor volume was drawn according to time and a significant difference was shown between the two groups (Figure 4C). These results provided further evidence that miR-675-5p plays a tumor suppressive role in NSCLC cancer.

GPR55 is a direct downstream target of miR-675-5p.

**Figure 5:**
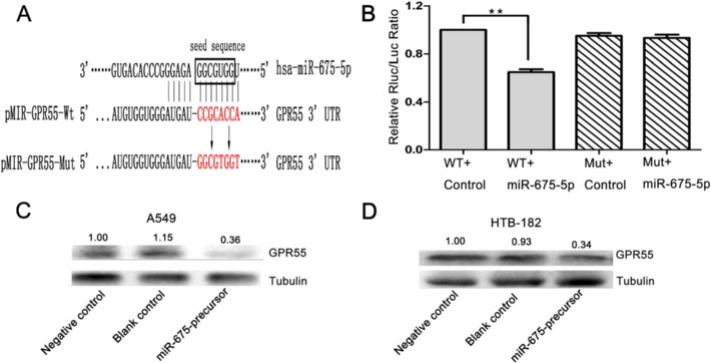
**GPR55 is a direct downstream target of miR-675-5p. (A)** Schematic of the construction of wild-type or mutant pGL3-GPR55 3′-UTR vectors is illustrated. **(B)** Relative luciferase activity was analyzed in A549 cells. Firefly luciferase vector was used as an internal control. **(C** and **D)** Related expression of GPR55 protein in A549 and HTB-182 cells treated with miR-675 precursor was determined by western blot analysis. Data were represented as the mean ± SEM of three independent experiments. Negative control: pGCsil-GFP Vector. *P < 0.05, **P < 0.01.

Next, we searched candidate target genes of miR-675-5p using publicly available databases. Among the candidates, GPR55 exhibited one of the highest prediction scores and the most complementary structure with miR-675-5p (Additional file 1: Table S2). Moreover up-regulation of GPR55 protein was found in various types cancer and high GPR55 expression is associated with more aggressive phenotypes [22-26]. Therefore, GPR55 was selected for further analysis (Figure 5A). To confirm whether GPR55 was a direct target of miR-675-5p, a dual-luciferase reporter system was used, employing co-transfection of miR-675 mimic and a luciferase reporter plasmid containing the 3′UTR of human GPR55. As shown in Figure 5B, the intensity of fluorescence after miR-675 mimic co-transfection was reduced significantly compared with the negative control; however, no significant variation in luciferase activity was observed for either the GPR55-Mut or the negative control with miR-675-mimic co-transfection. Thus, the luciferase assays revealed that miR-675-5p could bind to the GPR55 3′UTR, causing a significant decrease in luciferase activity compared with the negative control. In addition, western blot analysis showed that GPR55 protein expression was clearly decreased in A549 cells and HTB-182 cells transfected with LV-miR-675-5p-precursor (Figure 5 C and D). However, the protein levels of retinoblastoma (RB) protein, a known direct target of miR-675 in colorectal cancer and another target prediction of miR-675-5p, IkB kinase TBK1 protein, was also reported to be necessary in mediating KRAS-driven tumorigenicity in lung cancer remained unchanged in A549 cells and HTB-182 cells transfected with LV-miR-675-5p-precursor (Additional file 2: Figure S1 and Additional file 3: Figure S2) [18,19,27]. These results indicated that GPR55 was a direct downstream target for miR-675-5p in NSCLC cells.

Down-regulation of the expression of GPR55 influences the effects of miR-675 on NSCLC cells

**Figure 6:**
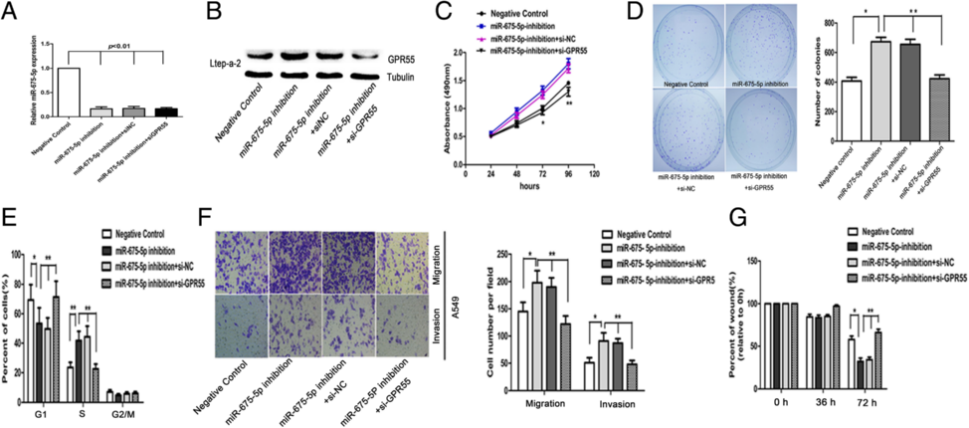
**Requirement of GPR55 for miR-675-5p induced suppression of NSCLC cell proliferation, migration and invasion. (A)** the level of miR-675-5p in the cells was significantly down-regulated after transfection with miR-675-5p inhibitor. **(B)** GPR55 was knockdowned in Ltep-a-2 miR-675-5p inhibition cells and analyzed by western blot analysis. Knockdown of GPR55 significantly inhibited proliferation **(C)**, inhibited colony formation **(D)**, induced cell cycle arrest at the G1/S phase **(E)**, inhibited migration and invasion **(F)**, and decreased the wound healing rate **(G)** in Ltep-a-2 cells. Data were represented as the mean ± SEM of three independent experiments. Negative control: pGC FU-RNAi-NC-LV. *P < 0.05, P* < 0.01.

To further ascertain whether GPR55 is a functional target of miR-675-5p, we transfected with LV-miR-675-5p inhibitor into Ltep-a-2 NSCLC cells, which have high endogenous miR-675-5p levels (Figure 1D). Compared with control cell group (scrambled sequence), the cells transfected with miR-675-5p inhibitor displayed higher expression of GPR55, whereas the cells with the co-transfection of both LV-miR-675-5p inhibitor and si-GPR55 exhibited lower GPR55 expression (Figure 6A). Interestingly, the cells transfected with LV-miR-675-5p inhibitor displayed higher proliferation, migration and invasion potential when compared with the cells transfected with both LV-miR-675-5p inhibitor and si-GPR55 (Figure 6B-F). We also transfected with si-GPR55 into A549 NSCLC cells, which have high endogenous GPR55 levels (new Additional file 4: Figure S3) and lower endogenous miR-675-5p levels (Figure 1D), and the expression of GPR55 in the cells determined by Western blotting. Compared with control cell group (non-specific control siRNA, si-NC), the cells transfected with si-GPR55 displayed lower expression of GPR55 and the cells transfected with si-GPR55 displayed lower growth rate compared with the cells transfected with si-NC. (Additional file 4: Figure S3). These observations suggest that the effects of miR-675-5p down-regulation on the promotion of cancer cell proliferation, migration and invasion could be diminished by si-GPR55. Therefore, GPR55 may mediate cell proliferation, migration and invasion of NSCLC induced by miR-675-5p.

MiR-675-5p inhibits the GPR55 signaling pathway.

**Figure 7:**
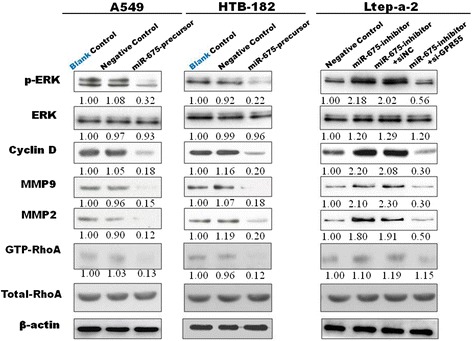
**miR-675-5p-mediated inhibition of the GPR55 signaling pathway.** In A549 and HTB-182 cells with miR-675-5p overexpression, the protein levels of p-ERK, Cyclin D, GTP-RhoA, MMP9 and MMP2 (left and middle, lane 3) were significantly decreased compared with the control. In Ltep-a-2 cells with miR-675-5p down-regulated expression, the protein levels of p-ERK, Cyclin D, GTP-RhoA, MMP9 and MMP2 (right, lane 2) were significantly increased compared with the negative control(right, lane 1) and si-GPR55 treatment abrogated the increased expression of these genes induced by miR-675-5p in the cells (right, lane 4). However, the expression of these genes has not changed in Ltep-a-2 cells (stable down-regulation of miR-675-5p) transfected with non-specific control siRNA (si-NC) (Figure 7, right, lane 3). Knockdown of GPR55 inhibited the expression of GPR55 and its main target genes, similar to miR-675-5p (right).

To explore whether miR-675-5p exerts its functions through the GPR55-ERK and/or GPR55-RhoA pathways that contribute to cancer proliferation, development and progression [28-31], we examined a number of the main GPR55 signaling downstream target genes, including phosphorylation of extracellular signal regulated kinase (p-ERK), ERK, Cyclin D1 protein (cyclin D1), active form of RhoA (GTP-RhoA), matrix metalloproteinase-2(MMP2), and MMP9. Expression of p-ERK, cyclin D1, GTP-RhoA, MMP2 and MMP9 were decreased in A549 and HTB-182 cells that stably overexpressed miR-675-5p (Figure 7, left and middle, lane 3). In contrast, expression of these genes was significantly up-regulated in Ltep-a-2 cells with stable down-regulation of miR-675-5p (Figure 7, right, lane 2) compared with negative control cell group (Negative control) (Figure 7, right, lane 1). Moreover, knockdown of the expression of GPR55 using GPR55-specific siRNA (si-GPR55) (Fig6A, lane 4) abrogated the effects induced by miR-675-5p down-regulation (Figure 7, right, lane 4). However, the expression of these genes did not change in Ltep-a-2 cells (stable down-regulation of miR-675-5p) transfected with non-specific control siRNA (si-NC) (Figure 7, right, lane 3). These data indicate that miR-675-5p inhibits GPR55 signaling in NSCLC, which involved tumor development and progression.

Up-regulation of GPR55 is inversely associated with down-regulation of miR-675-5p in clinical specimens of NSCLC.

**Figure 8:**
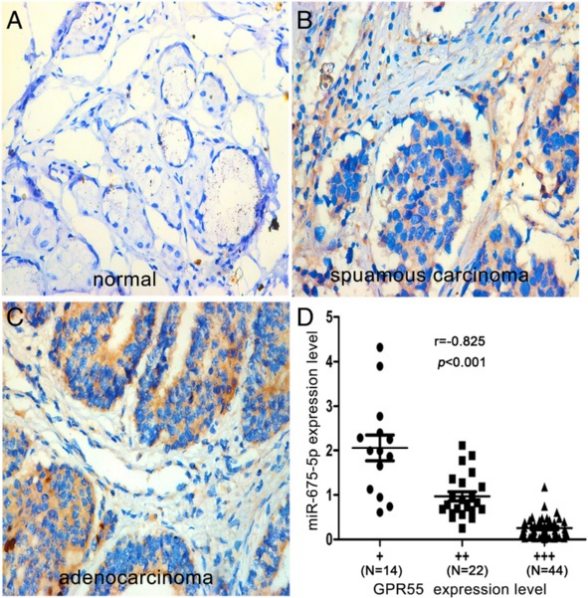
**Inverse correlation between the expression of GPR55 and miR-675-5p in clinical specimens of NSCLC. (A, B, C)** The expression of GPR55 was assessed in normal and NSCLC tissues using immunohistochemistry. The images were representative IHC of GPR55 in normal, squamous carcinoma, and adenocarcinoma, respectively. **(D)** A scatter diagram showed an inverse correlation between the expression of miR-675-5p and GPR55 in the same set of NSCLC tissue specimens. (original magnification ×400).

To further investigate the clinical significance of GPR55 expression, we examined GPR55 expression using immunohistochemical analysis on FFPEs of 80 NSCLC specimens. As shown in Figure 8A, GPR55 antibody exhibited negative or weak staining in alveolar epithelial cells of normal lung tissue. In contrast, GRP55 antibody displayed positive staining in tumor tissues with different intensity (Figure 8B and C). Furthermore, GRP55 expression was positively correlated with tumor, nodes and metastasis-classification (TNM) stage (P < 0.02) and lymph node metastasis of NSCLC (P < 0.01) (Additional file 5: Table S1). Taken together, GPR55 was frequently overexpressed in NSCLC and the elevated expression was positively associated with the progression of the disease.

To explore the relationship between miR-675-5p and GPR55 in clinical specimens, we compared GPR55 expression data from immunohistochemistry analysis with results of miR-675-5p expression level from qRT–PCR analysis on specimens of these NSCLC tissues. There was an inverse correlation between miR-675-5p and GPR55 expressions in these specimens (Figure 8D, R = -0.825, P < 0.001).

## Discussion

The abnormal expression of miRNAs has been reported in many types of cancer, and considerable attention is focused on understanding the role of miRNAs in the process of cancer development [32,33]. In the present study, we first found that miR-675-5p was frequently down-regulated in lung tumor tissues and the reduced miR-675-5p expression was closely related to advanced stage and lymph node metastasis of NSCLC. Furthermore, we demonstrated that miR-675-5p overexpression could suppress NSCLC cell proliferation, migration and invasion in vitro and tumor growth in vivo. In addition, we identified the pro-oncogenic GPR55 gene as a target of miR-675-5p. Therefore, miR-675-5p could be a novel tumor-suppressor miRNA, and its down-regulation might contribute to lung cancer progression and metastasis through regulating GPR55 function. The versatile functions of miR-675-5p in tumor cell proliferation, migration and invasion suggest its potential application as a prognostic predictor and cancer therapeutic target.

We have provided the following lines of evidence that miR-675-5p inhibits tumor growth, proliferation and migration in part by suppressing GPR55. (A) The mRNA levels of miR-675-5p were inversely correlated with GPR55 levels in NSCLC tissues. (B) Up-regulation of miR-675-5p significantly reduced GPR55 levels in NSCLC cells, whereas inhibition of miR-675-5p increased GPR55 levels. (C) Overexpression of miR-675-5p decreased the luciferase reporter activity of wild-type 3′- UTR but not mutant 3′-UTR of GPR55. (D) More importantly, the effects of miR-675-5p antagonists on cell proliferation, migration and invasion of NSCLC cells were abrogated by silencing of the GPR55 gene. These data support GPR55 as a downstream mediator of miR-675-5p function in NSCLC.

GPR55 is a G protein-coupled receptor with lipid-sensing properties and its up-regulation contributes to the aggressive behavior of various cancer types [22-26]. Andradas et al. found that most human cancer cell lines express detectable levels of GPR55 mRNA [28]. Higher GPR55 expression is associated with more aggressive phenotypes (higher histological grades and higher proliferative rates) in human breast tumors [28], pancreatic tumors [26] and glioblastomas [34]. Overexpression of GPR55 enhanced cell proliferation via extracellular signal-regulated protein kinase [28]. Ford et al found that the highly metastatic MDAMB-231 cell line expressed higher levels of GPR55 than the non-metastatic MCF-7 cell line and overexpression of GPR55 in MCF-7 cells enhanced their migration [30]. This suggests that signals from GPR55 could control not only cell proliferation but also cell migration and invasion. GPR55 activation resulted in increasing migration and invasion is likely through activation of RhoA that regulates cell morphology and mobility as well as membrane trafficking [31]. In the present study, we examined the expression of GPR55 signaling downstream target genes and found that expression of p-ERK, cyclin D1, MMP2 and MMP9 were decreased in NSCLC cells that stably overexpressed miR-675-5p. In contrast, expression of these genes was significantly up-regulated in NSCLC cells that stably expressed miR-675-5p inhibitor. Moreover, knockdown expression of GPR55 abrogated the effects induced by miR-675-5p -inhibitor. Cyclin D1 is a crucial mediator of G1 to S progression [35]. Up-regulation of cyclin Dl results in rapid growth of a subset of NSCLC [35]. Thus, down-regulation of cyclin D1 through inhibition of GPR55 could be a mechanism by which miR-675-5p suppresses cell proliferation and promotes cell cycle arrest at the G1 phase. MMPs are a family of enzymes that proteolytically degrade various components of the extracellular matrix [36]. High levels of certain MMPs are closely correlated with the invasive and metastatic potential of tumors [37,38]. Specifically, activated RhoA regulates tumor invasion of lung cancer cells by regulating gene transcription of MMP2 and MMP9 [39,40]. In contrast, blocking RhoA activity significantly inhibits MMP2 and MMP9 expression, tumor invasion, and lung metastasis [40,41]. Therefore, down-regulation of the RhoA-MMP2/9 axis through inhibition of GPR55 is one of the important mechanisms underlying miR-675-5p-mediated inhibition of NSCLC invasion and metastasis. These data indicate that miR-675-5p suppresses progression of NSCLC through inhibition of the versatile tumor-promoting GPR55. It is noteworthy that neither miR-675 nor GPR55 has been investigated in NSCLC. Our report revealed a novel miR-675-GPR55 axis in regulation of NSCLC.

GPR55 is identified as a target of miR-675-5p. However, the antioncogenic properties of miR-675-5p might not solely be explained by its ability to regulate a single gene alone, because a single miRNA can potentially regulate dozens to hundreds of genes in tumorigenesis [42]. Indeed, we identified at least 12 other potential targets of miR-675-5p using bioinformatic prediction analysis, including some tumor-related genes. For instance, TGFBI was recently proposed as a biologically relevant miR-675-5p target in prostate cancer [20]. TGFBI is an extracellular protein that promotes epithelial-mesenchymal transition and cancer metastasis [43]. Up-regulation of miR-675 in the prostate cancer cell line significantly decreased the level of TGFBI and repressed cell migration. Therefore, we cannot exclude the possibility that these candidate targets for miR-675-5p besides GPR55 could mediate tumor-suppressive function of miR-675-5p. We are exploring the correlation between miR-675-5p and other target candidates and determining whether miR-675-5p can biologically regulate the potential targets in a different study. Another example is pRB, a tumor suppressor that is targeted by miR-675 in colorectal cancer in which miR-675 acts as an oncogene [18,19] and IkB kinase TBK1 [27]. Intriguingly we did not observe any significant alteration at the protein levels of pRB and TBK1 by miR-675-5p in NSCLC cells (Additional file 2: Figure S1 and Additional file 3: Figure S2). These findings suggest miR-675 regulates its target genes and cancer cell behaviors in a cell or tissue type-specific in cancer. On the other hand, bioinformatic analysis suggests that the GPR55 may be targeted by more than 10 different miRNAs, implying that other miRNAs may also regulate function of GPR55 in lung tumorigenesis. For example, miR-3187-5p is one of the miRNAs that are predicted as candidates to regulate GPR55. Interestingly, miR-3187-5p is down-regulated in patients with bladder cancer and low miR-31 87-3p level is associated with tumor invasion [44]. Therefore, future studies to identify additional novel targets of miR-675-5p and other miRNAs that can also regulate GPR55 will allow us to have deep understanding of the mechanisms underlying the development and progression of NSCLC.

H19 has been shown to be the primary miRNA precursor of miR-675 in both human and mice and also been identified as a developmental reservoir of miR-675 that suppresses growth [45,46]. We evaluated the expression of miR-675-5p in NSCLC tissues and the corresponding normal lung tissues using quantitative reverse transcriptase PCR. There is no difference between the RNA levels of H19 in NSCLC tissues and that in the matching normal tissues (new Additional file 6: Figure S4). Because H19 is unchanged, we speculate down-regulation of miR-675-5p in NSCLC results from post-transcriptional regulation instead of transcriptional repression of miR-675-5p’s primary transcript H19. Downregulation of miR-675-5p may result from reduced conversion of H19 into pre-miR-675 and/or reduced conversion of pre-miR-675 into mature miR-675-5p.

## Conclusions

Carcinogenesis is a series of sequential events, including growth, proliferation, migration, and local invasion. Herein, we showed that miR-675-5p could suppress the carcinogenesis of NSCLC through inhibition of growth, proliferation, migration and invasion. Furthermore, our evidence suggests that miR-675-5p is a potential therapeutic target in NSCLC. Further studies are required to fully understand the detailed mechanisms of miR-675-5p in NSCLC carcinogenesis and as a potential therapeutic approach.

## Materials and Methods

### Ethical statement

Written informed consent was obtained from all participants, and the study protocol was approved by the ethics committee of Xiangya Hospital, Central South University (CSU). All mouse experiments were approved by the Animal Care and Use Committee (Permit#201403238) and conducted in accordance with the official recommendations of the Care and Use Laboratory Animals of Xiangya Hospital, CSU.

### Patient and tissue samples

Primary cancer tissues and paired adjacent non-tumor tissues were collected from 80 patients with NSCLC underwent lung resection at the Department of Surgery, Xiangya Hospital of Central South University from May 2011 to December 2013. Patients did not receive any preoperative cancer treatments, such as radiotherapy or chemotherapy. Each specimen was rapidly frozen in liquid nitrogen, and transferred to the -80°C refrigerator for subsequent experiments. The collected samples were confirmed by an experienced pathologist. The clinical data of NSCLC patients including tumor-node metastasis (TNM) staging were also collected.

### Cell lines and cell culture

Six NSCLC cell lines (HTB-182, A549, SPC-A-1, H1299, 95-D, Ltep-a-2) were obtained from the American Type Culture Collection. A normal human bronchial epithelial cell line (HBE), were purchased from the Institute of Biochemistry and Cell Biology of the Chinese Academy of Sciences (Shanghai, China). Cells were cultured in RPMI 1640 (GIBCO-BRL) medium supplemented with 10% fetal bovine serum (10% FBS), 100 U/ml penicillin, and 100 μg/ml streptomycin (Biyuntian, China) in humidified air at 37°C with 5% CO_2_.

### RNA extraction and qRT-PCR analyses

Total RNA was extracted from cell lines and frozen tumor specimens using Trizol reagent (Invitrogen, Carlsbad, CA, USA) according to the manufacturer’s protocol. qRT-PCR assays were performed to detect miR-675-5p and GPR55 expression using the PrimeScript RT reagent Kit and SYBR Premix Ex Taq (GeneCopoeia, USA) according to the manufacturer’s instructions. The relative level of miR-675-5p and GPR55 was determined by qRT-PCR using gene specific primers. U6 or β-actin was used as a normalization control. Levels of miR-675-5p and GPR55 were normalized to U6 and β-actin, respectively, to yield a 2^-ΔΔCt^ value for relative expression of each transcript. Experiments were repeated at least three times.

The RT reaction was carried out under the following conditions: 37°C for 60 min; 85°C for 5 min; and then held on 4°C. After the RT reaction, the complementary DNA products were diluted at1:10 and 2 μl of the diluted complementary DNA was used for subsequent qRT-PCR reactions. The qRT-PCR primers were designed as follows: miR-675-5p, Forward: 5′-UGGUGCGGAGAGGGCCCACAGUG-3′, Reverse: 5′-TGGTGTCGTGGAGTCG-3′. H19, Forward: 5′-CCGGACACAAAACCCTCTAGCT-3′, Reverse 5′-TGTTCCGATGGTGTCTTTGATG-3′; U6, Forward: 5′-CTCGCTTCGGCAGCACA-3′, Reverse: 5′-AACGCTTCACGAATTTGCGT-3′; Human GPR55, Forward: 5′-CTGCCTTGGTTCCACCATA-3′, Reverse: 5′-CCAGGATGCAGGTGAGTAAGA-3′. The qRT-PCR reaction was conducted at 95°C for 10 s and followed by 40 cycles of 95°C for 10 s, 60°C for 20 s and 72°C for 10 s in the ABI 7500 real-time PCR system (Applied Biosystems, CA, USA). The qRT-PCR results were analyzed and expressed as relative miRNA expression of CT (threshold cycle) value, which was then converted to fold changes.

### Vector construction and transfection

The hsa-miR-675-precursor sequence was constructed as follows: (Forward) hsa-miR-675-Age I-F ACCGGTGGAGGGCGAAGC, (Reverse) hsa-miR-675-EcoR I-R GAATTCAAAAACTCCTGAGAG. The sequence was amplified and cloned into the pGCsil-GFP Vector (GeneChem Co., Shanghai, China) to generate pGCsil-GFP-miR-675 and the pGCsil-GFP Vector only as negative control. The hsa-miR-675-5p-inhibition sequence was constructed as follows: (Forward) hsa-miR-675-5p-inhibition-Age I-F AATTCAAAAATGGTGCGGAGAGGGCCCACAGTG, (Reverse) hsa-miR-675-5p-inhibition-EcoR I-R CCGGCACTGTGGGCCCTCTCCGCACCATTTTTG. The sequence was amplified and cloned into the pGCsil-GFP Vector to generate pGCsil-GFP-miR-675-5p-inhibition. The non-silencing shRNA control sequences(TTCTCCGAACGTGTCACGT) was cloned into the pGCsil-GFP Vector as negative control (pGC FU-RNAi-NC-LV). Virus packaging, production and cell transfection were performed according to the manufacture’s protocol. The expression was validated by qRT-PCR. GPR55-siRNA (si-GPR55) and non-specific control siRNA (si-NC) were purchased from GeneChem, Shanghai, China.

### Cell proliferation, cell cycle and colony formation assays

Cell proliferation was monitored using Cell Proliferation Reagent Kit I (MTT) (Sigma). LV-miR-675-precursor, LV-negative control transfected A549 and HTB-182 or miR-675-5p-inhibition, or pGC FU-RNAi-NC-LV (Negative control) transfected Ltep-a-2 cells (3000/well) were allowed to grow in 96-well plates. Cell proliferation was documented every 24 h following the manufacturer’s protocol. Cell cycle analyses were performed using propidium iodide (Keygen, Nanjing, China). For cell cycle analysis, cells were seeded in 6-well plates at 2 × 10^5^ per well. Forty-eight hours after transfection, cells were fixed in 70% ethanol at 4°C for 24 hours and stained with 50 μg/mL propidium iodide (Keygen, Nanjing, China). The cell cycle distribution was analyzed by flow cytometry (Epics Altra, Beckman Coulter,USA). For the colony formation assay, LV-miR-675-5p-inhibition, LV-negative control transfected A549 and HTB-182 cells or miR-675-5p-inhibition, pGC FU-RNAi-NC-LV (Negative control) transfected Ltep-a-2 cells (1000/well) were allowed to grow in culture dish(10-cm) and maintained in media containing 10% FBS, replacing the medium every 4 days. After 10 days, cells were fixed with methanol and stained with 0.1% crystal violet (Biyuntian, Beijing, China). Visible colonies were manually counted. All experiments were performed in triplicate.

### In vitro cell migration and invasion assays

For the migration assays, 48 h after transfection, 2 × 10^4^ cells in serum-free media were placed into the upper chamber of an insert (8-μm pore size, BD). For the invasion assays, 1 × 10^5^ cells in serum-free media were placed into the upper chamber of an insert coated with Matrigel (BD, USA). Media containing 10% FBS were added to the lower chamber. After 48 hours of incubation, removing the cells remaining on the upper membrane with cotton wool, whereas the cells that had migrated or invaded through the membrane were stained with 0.1% crystal violet in methanol, imaged, and counted using an inverted microscope (Canon, Japan). For wound-healing assay, cells (1 × 10^6^ cells) were seeded in six-well plates, cultured overnight and transfected with miR-675-precursor, negative control or miR-675-5p-inhibition, pGC FU-RNAi-NC-LV (Negative control). Upon reaching the appropriate confluence, the cell layer was scratched with a sterile plastic tip and washed with culture medium twice and cultured again for up to 72 h with serum-free medium. Images were captured at different time points (0, 36 and 72 h) under a microscope to assess the rate of gap closure. Every experiment was repeated three times.

### Bioinformatics methods

Using bioinformatics software (DIANA TOOL, Targetscan, miRanda) to predict miR-675-5p potential target gene, combined with the literature and through the test screening, GPR55 was selected as a further object of study.

### Luciferase reporter assay

To construct a luciferase reporter vector, GPR55 3′-UTR fragment containing putative binding sites for miR-675-5p was amplified by PCR using the following primers: h-GPR55-F: CCGACTCGAGCGGAAGGACATCCTGTTCAG h-GPR55-R: ATTGCGGCCGCCTTTCCAGAACCTCCCAGTC, the PCR product was subcloned downstream of the luciferase gene in the pLUC Luciferase vector (Ruibo, Guangzhou,China) and named GPR55-3′-UTR-WT. For the mutated construct, using the following primers: h-GPR55-mut-F: GGATGATGGCGTGGTTCTTCACTGATGTGCTTC. h-GPR55 -mut-R: GTGAAGAACCACGCCATCATCCCACCACATCA.

A549 cells grown in 96-well plate were co-transfected with 50 nM miR-675 mimic or mimic negative control, 100 ng of GPR55-3′UTR-Wt or GPR55-3′UTR-Mut, using the Lipofectamie 2000 (Invitrogen, USA). After 48 h of transfection, luciferase activity was assessed according to the Dual-Luciferase Reporter Assay protocol (Promega, Madison, WI). Each experiment was repeated in triplicates.

### Western blotting and RhoA activation assay

Total protein was extracted by lysing cells in RIPA buffer containing protease inhibitor. Protein samples were separated by sodium dodecyl sulfate polyacrylamide gel electrophoresis (SDS-PAGE) and transferred onto polyvinylidene fluoride (PVDF) membranes. After blocking with 5% non-fat milk or 3% BSA in TBS-T, membranes were incubated with the primary antibody. The following antibodies were used: GPR55 (1:1000, Cayman, USA), ERK1 (1:2000, Santa Cruz, USA), p-ERK1/2 (1:2000, SantaCruz,USA), anti-cyclinD1 (1:2000), MMP2 (1:1000, Proteintech.,USA), MMP9 (1:1000, Proteintech Group,USA), RB(1:1000, Proteintech Group,USA), Tubulin (1:3000, Abcam, USA) and β-actin (1:3000, Bioword, Britain) TBK1(1:2000, Upstate Biotechnologies/Millipore, Billerica, MA) and goat-anti-rabbit IgG conjugated to horseradish peroxidase (HRP) (1:5000, Santa Cruz, USA), which was used as the secondary antibody. Cells were seeded on 10-cm cell culture plates, grown to 80% confluency, and serum starved overnight. To evaluate the activity of RhoA, the cells washed twice with ice-cold TBS and harvested on ice with 500 μl of 1X MLB lysis buffer (Upstate Biotechnology Inc.). Lysates were then clarified by centrifugation at 14,000 g for 10 min. The cell lysates were incubated with 30 μg of GST-RBD-agarose beads to precipitate GTP-bound RhoA. The beads were pelleted by brief centrifugation (12,000 g for 30 s), and washed 3 times with lysis buffer. Samples were denatured in sample buffer, boiled for 5 min at 90°C, and resolved using a 12% SDS-PAGE gel. Bound RhoA was detected by Western blot using a monoclonal anti-RhoA antibody, followed by secondary antibody incubation. Bands were visualized using the enhanced chemiluminescence kit (Santa Cruz Biotechnology, Santa Cruz, CA). Target signals were quantified by BandScan software (Bio-Rad, Hercules, CA) and defined as the ratio of target protein relative to β-actin or β-tubulin.

### NSCLC mouse model

Five-week-old BALB/C-nu nude male mice were used for animal studies, and all animals were maintained in the specific pathogen-free (SPF) conditions at our institution. For the in vivo tumor proliferation assay, 2 × 10^6^ A549 cells transfected with LV-miR-675-precursor or LV-negative control were injected subcutaneously into the nude mice (6 per group). Tumor growth was monitored by caliper measurement once or twice a week for at least 4 weeks. Tumor volume was calculated as follows: V = L × l^2^ × 0.5, where L and l represent the larger and the smaller tumor diameters, respectively. The mice were sacrificed after 4 weeks.

### Immunohistochemical staining

Formalin-fixed, paraffin-embedded tissues were cut into 4-μm sections. Following deparaffinization, sections were rehydrated and subjected to antigen retrieval by microwaving in 0.01 M sodium citrate (pH 6) for 10 minutes. Sections were incubated at 4°C overnight with monoclonal antibodies against GPR55 as mentioned above. Immunostaining was performed using ChemMate DAKO EnVision Detection Kit, Peroxidase/DAB, Rabbit/Mouse (code K 5007, DakoCytomation, Glostrup, Denmark) according to the manufacturer’s instructions. Subsequently, sections were counterstained with hematoxylin (Dako) and mounted in dimethyl benzene. Protein staining was evaluated under a light microscope at 400× magnification. Staining intensity was scored manually by two independent experienced pathologists as 0 = no staining, 1 = weak staining, 2 = moderate staining, and 3 = strong staining. Tumor cells in five fields were randomly selected and scored based on the percentage of positively stained cells (0-100%). The final IHC score was calculated by multiplying the intensity score with the percentage of positive cells.

### Statistical analysis

The relationship between miR-675-5p expression and clinicopathologic parameters was analyzed using the Pearson *X*^2^ test. Spearman’s correlation analysis was used to determine correlation between miR-675-5p and GPR55 expression. The differences between groups were analyzed using Student *t* test when there were only two groups, or assessed by one-way ANOVA when there were more than two groups. All statistical analyses were performed using the SPSS software (version 16.0, Chicago, IL). A two-tailed value of P < 0.05 was considered statistically significant.

## Additional files

Additional file 1: Table S2. The target prediction of miR-675-5p from websites DIANA TOOL Targetscan and miRanda,the GRP55 score is 0.916(from DIANA TOOL, miTG score), -0.64(from Targetscan),-0.4313 and -0.1271(from miRanda, mirSVR score )and other target genes prediction of miR-675-5p score has been included in Additional file 1: Table S2 or refer to DIANA TOOL, Targetscan and miRanda websites. Additional file 1: Table S2 79 targets prediction of miR-675-5p from websites DIANA TOOL. Threshold is set to 0.7. Additional file 2: Figure S1. The expression of retinoblastoma (RB) protein which a known direct target of miR-675 has not changed in A549 cells and HTB-182 cells transfected with LV-miR-675-5p-precursor. The expression of RB in the cells determined by Western blotting. RB expression was normalized using β-tubulin expression. Additional file 3: Figure S2. The expression of the non-canonical IkB kinase TBK1 protein which a target prediction of miR-675 has not changed in A549 cells and HTB-182 cells transfected with LV-miR-675-5p-precursor. The expression of TBK1 in the cells determined by Western blotting. TBK1 expression was normalized using β-tubulin expression. Additional file 4: Figure S3. Down-regulation of the expression of GPR55 inhibits the growth of on the A549 NSCLC cells. The expression of GPR55 protein in the A549 cells transfected with si-GPR55 determined by Western blotting. Compared with control cell group(non-specific control siRNA, si-NC), the cells transfected with si-GPR55 displayed lower expression of GPR55(left) and the cells transfected with si-GPR55 displayed lower growth rate compared with the cells transfected with si-NC(right). Additional file 5: Table S1. Correlations between GPR55 expression and clinicopathological variables in patients with NSCLC. Additional file 6: Figure S4. The expression of H19 in NSCLC tissues and the matching normal tissues were determined by qRT-PCR and normalized against an endogenous control (U6 RNA). There is no difference between the RNA levels of H19 in NSCLC tissues and that in the matching normal tissues. Data were represented as the mean±SEM of three independent experiments.

## References

[1] Jemal A, Bray F, Center MM, Ferlay J, Ward E, Forman D (2011). Global cancer statistics. CA Cancer J Clin.

[2] Anglim PP, Alonzo TA, Laird-Offringa IA (2008). DNA methylation-based biomarkers for early detection of non-small cell lung cancer: an update. Mol Cancer.

[3] Heist RS, Engelman JA (2012). SnapShot: non-small cell lung cancer. Cancer Cell.

[4] Lu Y, Govindan R, Wang L, Liu PY, Goodgame B, Wen W (2012). MicroRNA profiling and prediction of recurrence/relapse-free survival in stage I lung cancer. Carcinogenesis.

[5] Du L, Pertsemlidis A (2010). microRNAs and lung cancer: tumors and 22-mers. Cancer Metastasis Rev.

[6] Lin PY, Yu SL, Yang PC (2010). MicroRNA in lung cancer. Br J Cancer.

[7] Malleter M, Jacquot C, Rousseau B, Tomasoni C, Juge M, Pineau A (2012). miRNAs, a potential target in the treatment of Non-Small-Cell Lung Carcinomas. Gene.

[8] Li C, Nguyen HT, Zhuang Y, Lin Z, Flemington EK, Zhuo Y (2012). Comparative profiling of miRNA expression of lung adenocarcinoma cells in two-dimensional and three-dimensional cultures. Gene.

[9] Bartel DP (2004). MicroRNAs: genomics, biogenesis, mechanism, and function. Cell.

[10] Ambros V (2004). The functions of animal microRNAs. Nature.

[11] Sun K, Lai EC (2013). Adult-specific functions of animal microRNAs. Nat Rev Genet.

[12] Calin GA, Croce CM (2006). MicroRNA signatures in human cancers. Nat Rev Cancer.

[13] Kloosterman WP, Plasterk RH (2006). The diverse functions of microRNAs in animal development and disease. Dev Cell.

[14] Ventura A, Jacks T (2009). MicroRNAs and cancer: short RNAs go a long way. Cell.

[15] Shi Y, Wang Y, Luan W, Wang P, Tao T, Zhang J (2014). Long non-coding RNA H19 promotes glioma cell invasion by deriving miR-675. PLoS One.

[16] Zhuang M, Gao W, Xu J, Wang P, Shu Y (2014). The long non-coding RNA H19-derived miR-675 modulates human gastric cancer cell proliferation by targeting tumor suppressor RUNX1. Biochem Biophys Res Commun.

[17] Li H, Yu B, Li J, Su L, Yan M, Zhu Z (2014). Overexpression of lncRNA H19 enhances carcinogenesis and metastasis of gastric cancer. Oncotarget.

[18] Tsang WP, Ng EK, Ng SS, Jin H, Yu J, Sung JJ (2010). Oncofetal H19-derived miR-675 regulates tumor suppressor RB in human colorectal cancer. Carcinogenesis.

[19] Hernandez JM, Elahi A, Clark CW, Wang J, Humphries LA, Centeno B (2013). miR-675 mediates downregulation of Twist1 and Rb in AFP-secreting hepatocellular carcinoma. Ann Surg Oncol.

[20] Zhu M, Chen Q, Liu X, Sun Q, Zhao X, Deng R (2014). lncRNA H19/miR-675 axis represses prostate cancer metastasis by targeting TGFBI. FEBS J.

[21] Schmitz KJ, Helwig J, Bertram S, Sheu SY, Suttorp AC, Seggewiss J (2011). Differential expression of microRNA-675, microRNA-139-3p and microRNA-335 in benign and malignant adrenocortical tumours. J Clin Pathol.

[22] Oka S, Nakajima K, Yamashita A, Kishimoto S, Sugiura T (2007). Identification of GPR55 as a lysophosphatidylinositol receptor. Biochem Biophys Res Commun.

[23] Paul RK, Wnorowski A, Gonzalez-Mariscal I, Nayak SK, Pajak K, Moaddel R (2014). (R, R′)-4′-methoxy-1-naphthylfenoterol targets GPR55-mediated ligand internalization and impairs cancer cell motility. Biochem Pharmacol.

[24] Farsandaj N, Ghahremani MH, Ostad SN (2012). Role of cannabinoid and vanilloid receptors in invasion of human breast carcinoma cells. J Environ Pathol Toxicol Oncol.

[25] Perez-Gomez E, Andradas C, Flores JM, Quintanilla M, Paramio JM, Guzman M (2013). The orphan receptor GPR55 drives skin carcinogenesis and is upregulated in human squamous cell carcinomas. Oncogene.

[26] Pineiro R, Maffucci T, Falasca M (2011). The putative cannabinoid receptor GPR55 defines a novel autocrine loop in cancer cell proliferation. Oncogene.

[27] Barbie DA, Tamayo P, Boehm JS, Kim SY, Moody SE, Dunn IF (2009). Systematic RNA interference reveals that oncogenic KRAS-driven cancers require TBK1. Nature.

[28] Andradas C, Caffarel MM, Perez-Gomez E, Salazar M, Lorente M, Velasco G (2011). The orphan G protein-coupled receptor GPR55 promotes cancer cell proliferation via ERK. Oncogene.

[29] Hu G, Ren G, Shi Y (2011). The putative cannabinoid receptor GPR55 promotes cancer cell proliferation. Oncogene.

[30] Ford LA, Roelofs AJ, Anavi-Goffer S, Mowat L, Simpson DG, Irving AJ (2010). A role for L-alpha-lysophosphatidylinositol and GPR55 in the modulation of migration, orientation and polarization of human breast cancer cells. Br J Pharmacol.

[31] Henstridge CM, Balenga NA, Ford LA, Ross RA, Waldhoer M, Irving AJ (2009). The GPR55 ligand L-alpha-lysophosphatidylinositol promotes RhoA-dependent Ca2+ signaling and NFAT activation. FASEB J.

[32] Lu J, Getz G, Miska EA, Alvarez-Saavedra E, Lamb J, Peck D (2005). MicroRNA expression profiles classify human cancers. Nature.

[33] Landgraf P, Rusu M, Sheridan R, Sewer A, Iovino N, Aravin A (2007). A mammalian microRNA expression atlas based on small RNA library sequencing. Cell.

[34] Ross RA (2011). L-alpha-lysophosphatidylinositol meets GPR55: a deadly relationship. Trends Pharmacol Sci.

[35] Du B, Wang Z, Zhang X, Feng S, Wang G, He J (2014). MicroRNA-545 suppresses cell proliferation by targeting cyclin D1 and CDK4 in lung cancer cells. PLoS One.

[36] Zheng H, Takahashi H, Murai Y, Cui Z, Nomoto K, Niwa H (2006). Expressions of MMP-2, MMP-9 and VEGF are closely linked to growth, invasion, metastasis and angiogenesis of gastric carcinoma. Anticancer Res.

[37] Tsai CY, Wang CS, Tsai MM, Chi HC, Cheng WL, Tseng YH (2014). Interleukin-32 increases human gastric cancer cell invasion associated with tumor progression and metastasis. Clin Cancer Res.

[38] Fagan-Solis KD, Schneider SS, Pentecost BT, Bentley BA, Otis CN, Gierthy JF (2013). The RhoA pathway mediates MMP-2 and MMP-9-independent invasive behavior in a triple-negative breast cancer cell line. J Cell Biochem.

[39] Wu X, Liu T, Fang O, Leach LJ, Hu X, Luo Z (2014). miR-194 suppresses metastasis of non-small cell lung cancer through regulating expression of BMP1 and p27(kip1). Oncogene.

[40] Shieh JM, Wei TT, Tang YA, Huang SM, Wen WL, Chen MY (2012). Mitochondrial apoptosis and FAK signaling disruption by a novel histone deacetylase inhibitor, HTPB, in antitumor and antimetastatic mouse models. PLoS One.

[41] Dong QZ, Wang Y, Tang ZP, Fu L, Li QC, Wang ED (2013). Derlin-1 is overexpressed in non-small cell lung cancer and promotes cancer cell invasion via EGFR-ERK-mediated up-regulation of MMP-2 and MMP-9. Am J Pathol.

[42] Lim LP, Lau NC, Garrett-Engele P, Grimson A, Schelter JM, Castle J (2005). Microarray analysis shows that some microRNAs downregulate large numbers of target mRNAs. Nature.

[43] Shan B, Yao TP, Nguyen HT, Zhuo Y, Levy DR, Klingsberg RC (2008). Requirement of HDAC6 for transforming growth factor-beta1-induced epithelial-mesenchymal transition. J Biol Chem.

[44] Jiang X, Du L, Wang L, Li J, Liu Y, Zheng G (2015). Serum microRNA expression signatures identified from genome-wide microRNA profiling serve as novel noninvasive biomarkers for diagnosis and recurrence of bladder cancer. Int J Cancer.

[45] Cai X, Cullen BR (2007). The imprinted H19 noncoding RNA is a primary microRNA precursor. RNA.

[46] Keniry A, Oxley D, Monnier P, Kyba M, Dandolo L, Smits G (2012). The H19 lincRNA is a developmental reservoir of miR-675 that suppresses growth and Igf1r. Nat Cell Biol.

