# Supplementary material for: Down-regulation of miR-675-5p contributes to tumor progression and development by targeting pro-tumorigenic GPR55 in non-small cell lung cancer
Source: Mol Cancer. 2015 Apr 1;14:73. doi: 10.1186/s12943-015-0342-0 (PMC4392735; doi:10.1186/s12943-015-0342-0)
Supplement: Additional file 1: Table S2. — The target prediction of miR-675-5p from websites DIANA TOOL Targetscan and miRanda,the GRP55 score is 0.916(from DIANA TOOL, miTG score), -0.64(from Targetscan),-0.4313 and -0.1271(from miRanda, mirSVR score )and other target genes prediction of miR-675-5p score has been included in Additional file 1: Table S2 or refer to DIANA TOOL, Targetscan and miRanda websites. Additional file 1: Table S2 79 targets prediction of miR-675-5p from websites DIANA TOOL. Threshold is set to 0.7. [file 12943_2015_342_MOESM1_ESM.doc]

Additional file 6：The target prediction of miR-675-5p from websites DIANA TOOL Targetscan and miRanda，the GRP55 score is 0.916(from DIANA TOOL, miTG score), -0.64(from Targetscan),-0.4313 and -0.1271(from miRanda, mirSVR score )and other target genes prediction of miR-675-5p score has been included in Additional file 6 Table S2 or refer to DIANA TOOL, Targetscan and miRanda websites.

Table S2 79 targets prediction of miR-675-5p from websites DIANA TOOL. Threshold is set to 0.7.

| **Transcript Id** | **Gene Id(name)** | **Mirna Name** | **miTG score** |
| --- | --- | --- | --- |
| ENST00000460277 | ENSG00000117262 (GPR89A) | hsa-miR-675-5p | 0.997 |
| UTR3 | 1:145765162-145765190 | 0.105321322 |  |
| UTR3 | 1:145765040-145765068 | 0.002984072 |  |
| ENST00000469076 | ENSG00000040487 (PQLC2) | hsa-miR-675-5p | 0.992 |
| UTR3 | 1:19653087-19653115 | 0.085204796 |  |
| UTR3 | 1:19655144-19655172 | 0.001625495 |  |
| UTR3 | 1:19655464-19655492 | 0.001783472 |  |
| ENST00000571371 | ENSG00000154975 (CA10) | hsa-miR-675-5p | 0.989 |
| UTR3 | 17:49710903-49710931 | 0.084006024 |  |
| ENST00000583465 | ENSG00000173826 (KCNH6) | hsa-miR-675-5p | 0.987 |
| UTR3 | 17:61611507-61611535 | 0.07892698 |  |
| UTR3 | 17:61623043-61623071 | 0.002539286 |  |
| ENST00000574448 | ENSG00000103316 (CRYM) | hsa-miR-675-5p | 0.984 |
| UTR3 | 16:21279037;21279868-21279058;21279874 | 0.077983795 |  |
| ENST00000443805 | ENSG00000115657 (ABCB6) | hsa-miR-675-5p | 0.977 |
| UTR3 | 2:220075468-220075496 | 0.028281254 |  |
| UTR3 | 2:220075122-220075150 | 0.043501224 |  |
| ENST00000325074 | ENSG00000159216 (RUNX1) | hsa-miR-675-5p | 0.969 |
| UTR3 | 21:36164182-36164210 | 0.001303038 |  |
| UTR3 | 21:36163929-36163957 | 0.063120158 |  |
| UTR3 | 21:36162904-36162932 | 0.000796232 |  |
| UTR3 | 21:36162271-36162299 | 0.001797093 |  |
| ENST00000515351 | ENSG00000156453 (PCDH1) | hsa-miR-675-5p | 0.967 |
| UTR3 | 5:141244803-141244831 | 0.00322342 |  |
| UTR3 | 5:141244384-141244412 | 0.001842119 |  |
| UTR3 | 5:141243897-141243925 | 0.003564226 |  |
| UTR3 | 5:141243757-141243785 | 0.001985931 |  |
| UTR3 | 5:141242921-141242949 | 0.004821835 |  |
| UTR3 | 5:141242823-141242851 | 0.050399899 |  |
| ENST00000327979 | ENSG00000042062 (FAM65C) | hsa-miR-675-5p | 0.949 |
| UTR3 | 20:49203022-49203050 | 0.058510856 |  |
| ENST00000326571 | ENSG00000180269 (GPR139) | hsa-miR-675-5p | 0.948 |
| UTR3 | 16:20043683-20043711 | 0.005752809 |  |
| UTR3 | 16:20043383-20043411 | 0.008136402 |  |
| UTR3 | 16:20043236-20043264 | 0.044183858 |  |
| ENST00000492555 | ENSG00000168268 (NT5DC2) | hsa-miR-675-5p | 0.945 |
| UTR3 | 3:52558695-52558723 | 0.057305755 |  |
| ENST00000578101 | ENSG00000177731 (FLII) | hsa-miR-675-5p | 0.932 |
| UTR3 | 17:18157438-18157466 | 0.004379951 |  |
| UTR3 | 17:18152124-18152152 | 0.00100153 |  |
| UTR3 | 17:18149644-18149672 | 0.04490926 |  |
| UTR3 | 17:18148310-18148338 | 0.003184034 |  |
| ENST00000554702 | ENSG00000171988 (JMJD1C) | hsa-miR-675-5p | 0.932 |
| UTR3 | 10:64976987-64977015 | 0.046684146 |  |
| UTR3 | 10:64973408-64973436 | 0.002754351 |  |
| UTR3 | 10:64967808-64967836 | 0.002663469 |  |
| UTR3 | 10:64967064-64967092 | 0.001327695 |  |
| ENST00000392040 | ENSG00000135898 (GPR55) | hsa-miR-675-5p | 0.916 |
| UTR3 | 2:231774619-231774647 | 0.049748849 |  |
| ENST00000419047 | ENSG00000121068 (TBX2) | hsa-miR-675-5p | 0.914 |
| UTR3 | 17:59480487-59480515 | 0.049237837 |  |
| CDS | 17:59477760-59477788 | 0.002205176 |  |
| ENST00000427593 | ENSG00000163060 (TEKT4) | hsa-miR-675-5p | 0.912 |
| UTR3 | 2:95539054-95539082 | 0.048167871 |  |
| CDS | 2:95537405-95537433 | 0.009169288 |  |
| ENST00000394900 | ENSG00000149476 (DAK) | hsa-miR-675-5p | 0.906 |
| UTR3 | 11:61114308-61114336 | 0.046269833 |  |
| UTR3 | 11:61115062-61115090 | 0.001423721 |  |
| ENST00000322272 | ENSG00000239697 (TNFSF12) | hsa-miR-675-5p | 0.901 |
| UTR3 | 17:7460574-7460602 | 0.046879148 |  |
| ENST00000534480 | ENSG00000167861 (HID1) | hsa-miR-675-5p | 0.895 |
| UTR3 | 17:72958005-72958033 | 0.004679422 |  |
| UTR3 | 17:72954283-72954311 | 0.002323057 |  |
| UTR3 | 17:72950323-72950351 | 0.000937262 |  |
| UTR3 | 17:72948025-72948053 | 0.037766094 |  |
| ENST00000490910 | ENSG00000101343 (CRNKL1) | hsa-miR-675-5p | 0.888 |
| UTR3 | 20:20028421-20028449 | 0.043435037 |  |
| UTR3 | 20:20016873-20016901 | 0.00107166 |  |
| ENST00000454337 | ENSG00000261732 (LA16c-431H6.6) | hsa-miR-675-5p | 0.876 |
| UTR3 | 16:1705897-1705925 | 0.026518729 |  |
| UTR3 | 16:1724119-1724147 | 0.009179049 |  |
| UTR3 | 16:1736306-1736334 | 0.006337491 |  |
| CDS | 16:1682302-1682330 | 0.005899741 |  |
| ENST00000495831 | ENSG00000136286 (MYO1G) | hsa-miR-675-5p | 0.872 |
| UTR3 | 7:45015187-45015215 | 0.034918221 |  |
| UTR3 | 7:45009375-45009403 | 0.001161218 |  |
| UTR3 | 7:45009335-45009363 | 0.000360625 |  |
| UTR3 | 7:45002481;45002569-45002494;45002583 | 0.00287632 |  |
| UTR3 | 7:45002326-45002354 | 0.001621599 |  |
| UTR3 | 7:45002317-45002345 | 0.001147944 |  |
| ENST00000437868 | ENSG00000125505 (MBOAT7) | hsa-miR-675-5p | 0.869 |
| UTR3 | 19:54684827;54687404-54684850;54687408 | 0.0163563 |  |
| UTR3 | 19:54682502-54682530 | 0.003781114 |  |
| UTR3 | 19:54677313-54677341 | 0.021466956 |  |
| ENST00000555609 | ENSG00000258947 (TUBB3) | hsa-miR-675-5p | 0.862 |
| UTR3 | 16:90001474-90001502 | 0.040698493 |  |
| ENST00000523609 | ENSG00000123908 (AGO2) | hsa-miR-675-5p | 0.853 |
| UTR3 | 8:141549453-141549481 | 0.002708211 |  |
| UTR3 | 8:141542157-141542185 | 0.036719764 |  |
| ENST00000474964 | ENSG00000073111 (MCM2) | hsa-miR-675-5p | 0.851 |
| UTR3 | 3:127325456-127325484 | 0.039144549 |  |
| ENST00000560106 | ENSG00000100889 (PCK2) | hsa-miR-675-5p | 0.844 |
| UTR3 | 14:24567577;24567684-24567596;24567692 | 0.038252729 |  |
| ENST00000346199 | ENSG00000053438 (NNAT) | hsa-miR-675-5p | 0.843 |
| UTR3 | 20:36151455-36151483 | 0.003542324 |  |
| UTR3 | 20:36151518-36151546 | 0.010095328 |  |
| UTR3 | 20:36151578-36151606 | 0.002214112 |  |
| UTR3 | 20:36152061-36152089 | 0.022271806 |  |
| ENST00000453505 | ENSG00000240849 (TMEM189) | hsa-miR-675-5p | 0.84 |
| UTR3 | 20:48746199-48746227 | 0.004887019 |  |
| UTR3 | 20:48746092-48746120 | 0.032884674 |  |
| ENST00000478500 | ENSG00000107242 (PIP5K1B) | hsa-miR-675-5p | 0.839 |
| UTR3 | 9:71623410-71623438 | 0.037696382 |  |
| ENST00000342784 | ENSG00000105137 (SYDE1) | hsa-miR-675-5p | 0.839 |
| UTR3 | 19:15225003-15225031 | 0.002425154 |  |
| UTR3 | 19:15225722-15225750 | 0.032834424 |  |
| CDS | 19:15220528-15220556 | 0.005814216 |  |
| CDS | 19:15224670-15224698 | 0.009607621 |  |
| ENST00000592595 | ENSG00000141639 (MAPK4) | hsa-miR-675-5p | 0.838 |
| UTR3 | 18:48255781-48255809 | 0.032229706 |  |
| UTR3 | 18:48256903-48256931 | 0.001369058 |  |
| UTR3 | 18:48257100-48257128 | 0.002460733 |  |
| UTR3 | 18:48257472-48257500 | 0.00146581 |  |
| ENST00000295666 | ENSG00000163453 (IGFBP7) | hsa-miR-675-5p | 0.829 |
| UTR3 | 4:57897072-57897100 | 0.036481837 |  |
| ENST00000502426 | ENSG00000108846 (ABCC3) | hsa-miR-675-5p | 0.824 |
| UTR3 | 17:48753680-48753708 | 0.000579216 |  |
| UTR3 | 17:48764999-48765027 | 0.035400058 |  |
| ENST00000560255 | ENSG00000259642 (C15orf37) | hsa-miR-675-5p | 0.824 |
| UTR3 | 15:80215767-80215795 | 0.035905782 |  |
| ENST00000167586 | ENSG00000186847 (KRT14) | hsa-miR-675-5p | 0.822 |
| CDS | 17:39741228-39741256 | 0.033149726 |  |
| CDS | 17:39739844-39739872 | 0.005821972 |  |
| CDS | 17:39738755-39738783 | 0.056299024 |  |
| CDS | 17:39738695-39738723 | 0.008907014 |  |
| ENST00000490715 | ENSG00000007341 (ST7L) | hsa-miR-675-5p | 0.821 |
| UTR3 | 1:113066172-113066200 | 0.035608697 |  |
| ENST00000483281 | ENSG00000204231 (RXRB) | hsa-miR-675-5p | 0.817 |
| UTR3 | 6:33165662-33165690 | 0.035199384 |  |
| ENST00000503548 | ENSG00000163945 (UVSSA) | hsa-miR-675-5p | 0.815 |
| UTR3 | 4:1378307-1378335 | 0.001270711 |  |
| UTR3 | 4:1378459-1378487 | 0.000695514 |  |
| UTR3 | 4:1380990-1381018 | 0.002092971 |  |
| UTR3 | 4:1381488-1381516 | 0.030860073 |  |
| ENST00000359888 | ENSG00000196960 (AL117340.1) | hsa-miR-675-5p | 0.814 |
| UTR3 | 10:31651569-31651597 | 0.034815297 |  |
| ENST00000447947 | ENSG00000196644 (GPR89C) | hsa-miR-675-5p | 0.8 |
| CDS | 1:145923272-145923300 | 0.097313064 |  |
| ENST00000493509 | ENSG00000126952 (NXF5) | hsa-miR-675-5p | 0.8 |
| UTR3 | X:101093359;101093762-101093382;101093766 | 0.033350782 |  |
| ENST00000361127 | ENSG00000198799 (LRIG2) | hsa-miR-675-5p | 0.798 |
| UTR3 | 1:113666826-113666854 | 0.032406896 |  |
| CDS | 1:113616192-113616220 | 0.004742032 |  |
| ENST00000314163 | ENSG00000188092 (GPR89B) | hsa-miR-675-5p | 0.791 |
| CDS | 1:147464978-147465006 | 0.094553124 |  |
| ENST00000574426 | ENSG00000132361 (CLUH) | hsa-miR-675-5p | 0.789 |
| UTR3 | 17:2595105-2595133 | 0.030508087 |  |
| CDS | 17:2606711-2606739 | 0.00972882 |  |
| ENST00000556726 | ENSG00000015133 (CCDC88C) | hsa-miR-675-5p | 0.788 |
| UTR3 | 14:91749676-91749704 | 0.031083306 |  |
| UTR3 | 14:91739506-91739534 | 0.001082175 |  |
| ENST00000292357 | ENSG00000187800 (PEAR1) | hsa-miR-675-5p | 0.777 |
| UTR3 | 1:156886001-156886029 | 0.004540063 |  |
| UTR3 | 1:156886165-156886193 | 0.026522453 |  |
| ENST00000397621 | ENSG00000127585 (FBXL16) | hsa-miR-675-5p | 0.777 |
| UTR3 | 16:743744-743772 | 0.02727742 |  |
| UTR3 | 16:743655-743683 | 0.002096925 |  |
| UTR3 | 16:743579-743607 | 0.001740168 |  |
| ENST00000582022 | ENSG00000161526 (SAP30BP) | hsa-miR-675-5p | 0.774 |
| UTR3 | 17:73700888;73702088-73700894;73702109 | 0.008585068 |  |
| UTR3 | 17:73702093-73702121 | 0.008102321 |  |
| UTR3 | 17:73702550-73702578 | 0.010956405 |  |
| UTR3 | 17:73702771-73702799 | 0.002070273 |  |
| UTR3 | 17:73703442-73703470 | 0.001126414 |  |
| ENST00000286201 | ENSG00000155760 (FZD7) | hsa-miR-675-5p | 0.77 |
| CDS | 2:202900699-202900727 | 0.088796545 |  |
| ENST00000300843 | ENSG00000007047 (MARK4) | hsa-miR-675-5p | 0.767 |
| UTR3 | 19:45805905-45805933 | 0.030199875 |  |
| ENST00000512844 | ENSG00000153922 (CHD1) | hsa-miR-675-5p | 0.766 |
| UTR3 | 5:98192262-98192290 | 0.030056327 |  |
| ENST00000397412 | ENSG00000007545 (CRAMP1L) | hsa-miR-675-5p | 0.763 |
| UTR3 | 16:1724119-1724147 | 0.02345029 |  |
| UTR3 | 16:1726891-1726919 | 0.001732706 |  |
| CDS | 16:1682302-1682330 | 0.00939926 |  |
| CDS | 16:1705897-1705925 | 0.012962367 |  |
| ENST00000355633 | ENSG00000105991 (HOXA1) | hsa-miR-675-5p | 0.761 |
| UTR3 | 7:27134364-27134392 | 0.02963799 |  |
| ENST00000298298 | ENSG00000165511 (C10orf25) | hsa-miR-675-5p | 0.761 |
| UTR3 | 10:45493231-45493259 | 0.0296595 |  |
| ENST00000593066 | ENSG00000130811 (EIF3G) | hsa-miR-675-5p | 0.76 |
| UTR3 | 19:10229391;10229544-10229403;10229559 | 0.029513605 |  |
| ENST00000451271 | ENSG00000163702 (IL17RC) | hsa-miR-675-5p | 0.756 |
| UTR3 | 3:9970040-9970068 | 0.001324325 |  |
| UTR3 | 3:9975034-9975062 | 0.027892294 |  |
| ENST00000445478 | ENSG00000070495 (JMJD6) | hsa-miR-675-5p | 0.754 |
| UTR3 | 17:74712051-74712079 | 0.02900624 |  |
| ENST00000341900 | ENSG00000144355 (DLX1) | hsa-miR-675-5p | 0.753 |
| UTR3 | 2:172952926-172952954 | 0.028914017 |  |
| ENST00000586604 | ENSG00000141622 (RNF165) | hsa-miR-675-5p | 0.753 |
| UTR3 | 18:44027596-44027624 | 0.008353623 |  |
| UTR3 | 18:44037076-44037104 | 0.016643538 |  |
| UTR3 | 18:44040138-44040166 | 0.003416003 |  |
| UTR3 | 18:44041542-44041570 | 0.000495733 |  |
| ENST00000301904 | ENSG00000168077 (SCARA3) | hsa-miR-675-5p | 0.75 |
| UTR3 | 8:27530284-27530312 | 0.002996804 |  |
| UTR3 | 8:27530381-27530409 | 0.02491214 |  |
| CDS | 8:27516461-27516489 | 0.004033621 |  |
| ENST00000264071 | ENSG00000104833 (TUBB4A) | hsa-miR-675-5p | 0.747 |
| UTR3 | 19:6494897-6494925 | 0.021943684 |  |
| CDS | 19:6495867-6495895 | 0.028521755 |  |
| ENST00000086933 | ENSG00000063515 (GSC2) | hsa-miR-675-5p | 0.741 |
| UTR3 | 22:19136150-19136178 | 0.02255161 |  |
| CDS | 22:19137295-19137323 | 0.024226087 |  |
| ENST00000381128 | ENSG00000129221 (AIPL1) | hsa-miR-675-5p | 0.739 |
| UTR3 | 17:6328861-6328889 | 0.00203831 |  |
| UTR3 | 17:6327344-6327372 | 0.001768513 |  |
| CDS | 17:6337397;6338271-6337418;6338277 | 0.074282904 |  |
| ENST00000573150 | ENSG00000181523 (SGSH) | hsa-miR-675-5p | 0.739 |
| UTR3 | 17:78184514-78184542 | 0.027054162 |  |
| CDS | 17:78190931-78190959 | 0.003369392 |  |
| ENST00000450765 | ENSG00000115568 (ZNF142) | hsa-miR-675-5p | 0.727 |
| UTR3 | 2:219509492-219509520 | 0.012006208 |  |
| UTR3 | 2:219508240-219508268 | 0.00058962 |  |
| UTR3 | 2:219506855-219506883 | 0.004899953 |  |
| UTR3 | 2:219503462-219503490 | 0.001591686 |  |
| UTR3 | 2:219503379-219503407 | 0.003489697 |  |
| UTR3 | 2:219503195-219503223 | 0.004169807 |  |
| ENST00000331710 | ENSG00000183735 (TBK1) | hsa-miR-675-5p | 0.721 |
| UTR3 | 12:64895577-64895605 | 0.026251395 |  |
| ENST00000588558 | ENSG00000141349 (G6PC3) | hsa-miR-675-5p | 0.719 |
| UTR3 | 17:42152745-42152773 | 0.001358065 |  |
| UTR3 | 17:42153515-42153543 | 0.024740746 |  |
| ENST00000503143 | ENSG00000251380 (C5orf20) | hsa-miR-675-5p | 0.714 |
| UTR3 | 5:134780073-134780101 | 0.025685186 |  |
| ENST00000371494 | ENSG00000162383 (SLC1A7) | hsa-miR-675-5p | 0.709 |
| CDS | 1:53569231;53571404-53569240;53571422 | 0.037610695 |  |
| CDS | 1:53554635;53555472-53554651;53555483 | 0.036090981 |  |
| ENST00000543856 | ENSG00000172828 (CES3) | hsa-miR-675-5p | 0.706 |
| UTR3 | 16:67007953-67007981 | 0.002008918 |  |
| UTR3 | 16:67008707-67008735 | 0.003577336 |  |
| UTR3 | 16:67008835-67008863 | 0.019404807 |  |
| ENST00000555997 | ENSG00000185650 (ZFP36L1) | hsa-miR-675-5p | 0.705 |
| UTR3 | 14:69256786-69256814 | 0.019856421 |  |
| UTR3 | 14:69256525-69256553 | 0.005118416 |  |
| ENST00000564275 | ENSG00000168447 (SCNN1B) | hsa-miR-675-5p | 0.705 |
| UTR3 | 16:23390044-23390072 | 0.004512794 |  |
| UTR3 | 16:23391439-23391467 | 0.020443434 |  |
| ENST00000455477 | ENSG00000158089 (GALNT14) | hsa-miR-675-5p | 0.704 |
| UTR3 | 2:31181314;31189070-31181330;31189081 | 0.021013458 |  |
| UTR3 | 2:31178558-31178586 | 0.003840552 |  |
| ENST00000290497 | ENSG00000168907 (PLA2G4F) | hsa-miR-675-5p | 0.703 |
| UTR3 | 15:42438349-42438377 | 0.024768468 |  |
| ENST00000549147 | ENSG00000135441 (BLOC1S1) | hsa-miR-675-5p | 0.703 |
| UTR3 | 12:56113309-56113337 | 0.02482539 |  |
| ENST00000373736 | ENSG00000084628 (NKAIN1) | hsa-miR-675-5p | 0.702 |
| UTR3 | 1:31654309-31654337 | 0.001748797 |  |
| UTR3 | 1:31654022-31654050 | 0.022947845 |  |
| ENST00000381655 | ENSG00000132932 (ATP8A2) | hsa-miR-675-5p | 0.701 |
| UTR3 | 13:26594737-26594765 | 0.020570834 |  |
| UTR3 | 13:26598683-26598711 | 0.001557908 |  |
| CDS | 13:26043182-26043210 | 0.011332026 |  |
| ENST00000566266 | ENSG00000141013 (GAS8) | hsa-miR-675-5p | 0.7 |
| UTR3 | 16:90097863-90097891 | 0.004348735 |  |
| UTR3 | 16:90099138-90099166 | 0.004584297 |  |
| UTR3 | 16:90106917;90108878-90106917;90108905 | 0.002395444 |  |
| UTR3 | 16:90110495-90110523 | 0.013204427 |  |
|  |  |  |  |
|  |  |  |  |
|  |  |  |  |
|  |  |  |  |
|  |  |  |  |
|  |  |  |  |
|  |  |  |  |
|  |  |  |  |
|  |  |  |  |
|  |  |  |  |
|  |  |  |  |
|  |  |  |  |
|  |  |  |  |
