# Supplementary figures and images for: Down-regulation of miR-675-5p contributes to tumor progression and development by targeting pro-tumorigenic GPR55 in non-small cell lung cancer
Source: Mol Cancer. 2015 Apr 1;14:73. doi: 10.1186/s12943-015-0342-0 (PMC4392735; doi:10.1186/s12943-015-0342-0)

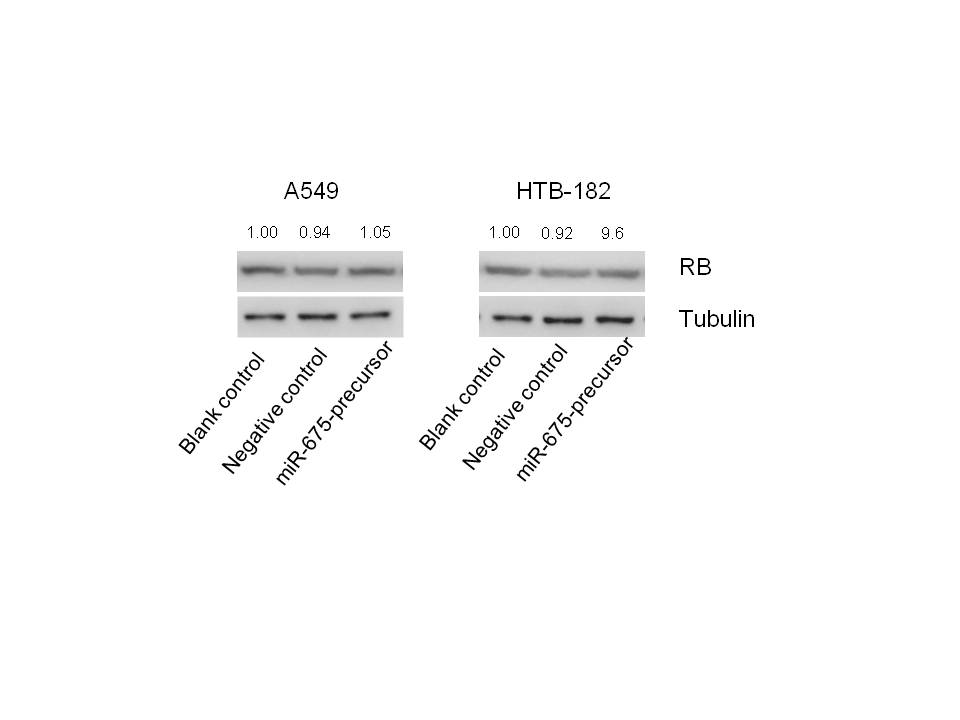

Supplement: Additional file 2: Figure S1. — The expression of retinoblastoma (RB) protein which a known direct target of miR-675 has not changed in A549 cells and HTB-182 cells transfected with LV-miR-675-5p-precursor. The expression of RB in the cells determined by Western blotting. RB expression was normalized using β-tubulin expression. [file 12943_2015_342_MOESM2_ESM.jpeg]

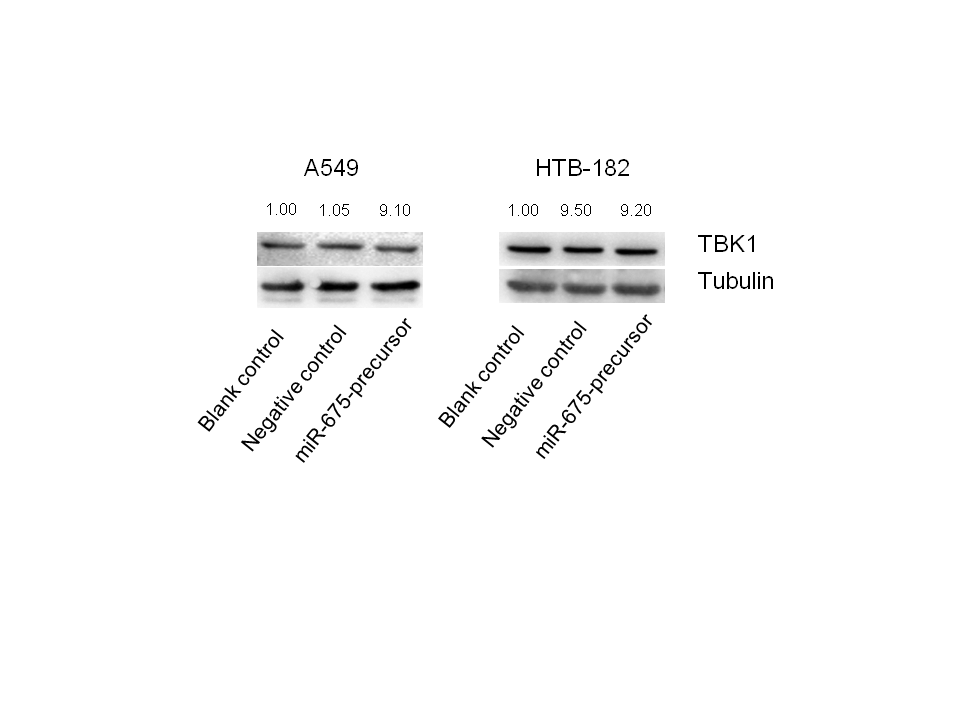

Supplement: Additional file 3: Figure S2. — The expression of the non-canonical IkB kinase TBK1 protein which a target prediction of miR-675 has not changed in A549 cells and HTB-182 cells transfected with LV-miR-675-5p-precursor. The expression of TBK1 in the cells determined by Western blotting. TBK1 expression was normalized using β-tubulin expression. [file 12943_2015_342_MOESM3_ESM.tiff]

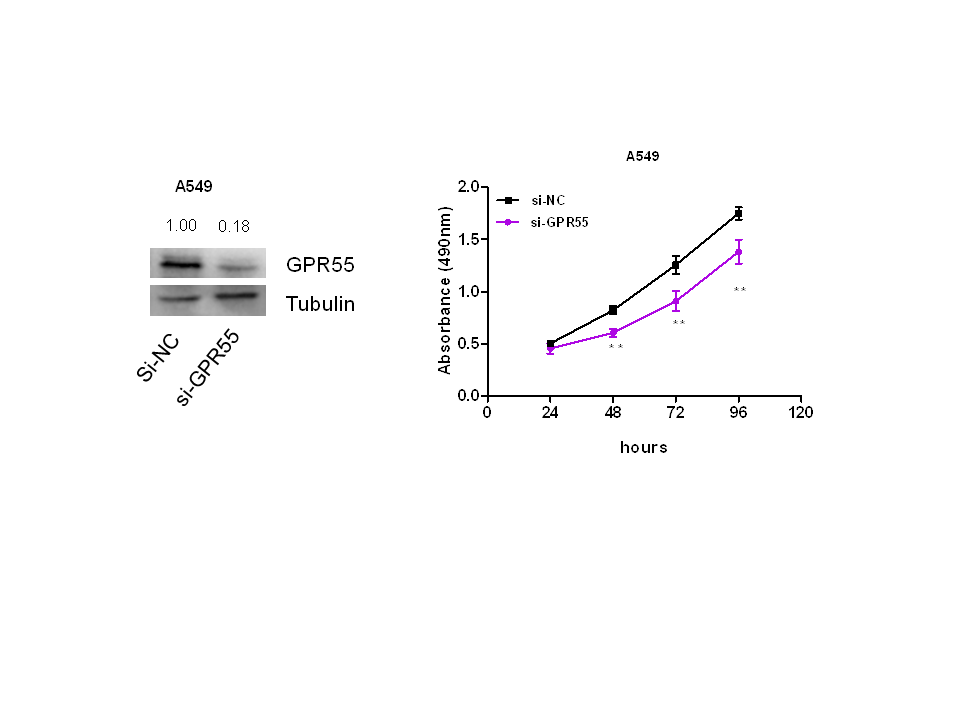

Supplement: Additional file 4: Figure S3. — Down-regulation of the expression of GPR55 inhibits the growth of on the A549 NSCLC cells. The expression of GPR55 protein in the A549 cells transfected with si-GPR55 determined by Western blotting. Compared with control cell group(non-specific control siRNA, si-NC), the cells transfected with si-GPR55 displayed lower expression of GPR55(left) and the cells transfected with si-GPR55 displayed lower growth rate compared with the cells transfected with si-NC(right). [file 12943_2015_342_MOESM4_ESM.tiff]

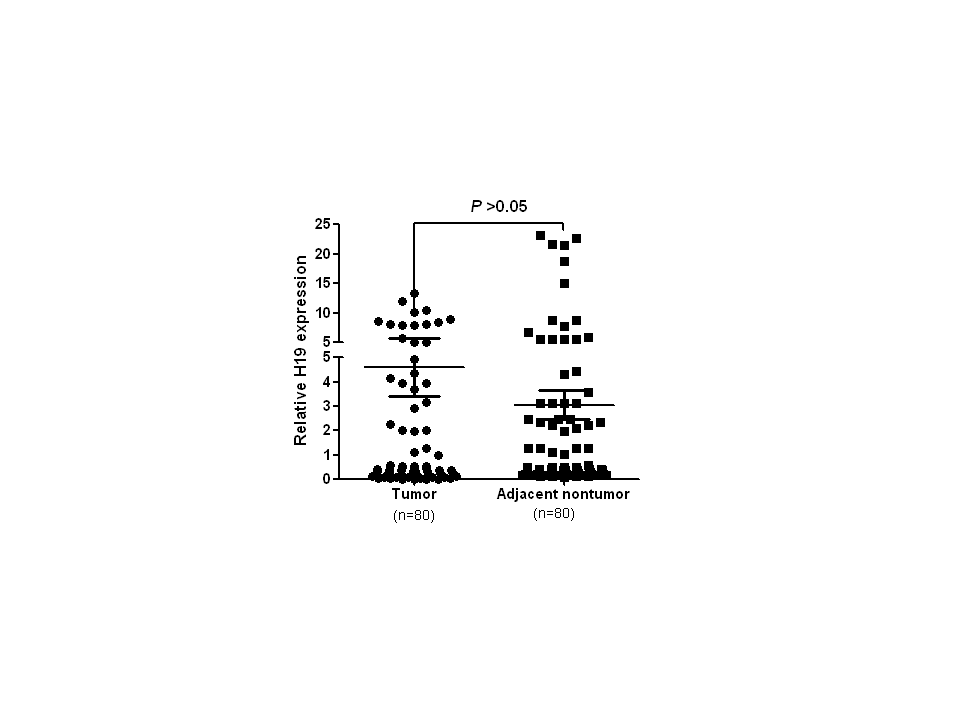

Supplement: Additional file 6: Figure S4. — The expression of H19 in NSCLC tissues and the matching normal tissues were determined by qRT-PCR and normalized against an endogenous control (U6 RNA). There is no difference between the RNA levels of H19 in NSCLC tissues and that in the matching normal tissues. Data were represented as the mean±SEM of three independent experiments. [file 12943_2015_342_MOESM6_ESM.tiff]
