# Supplementary material for: Down-regulation of miR-675-5p contributes to tumor progression and development by targeting pro-tumorigenic GPR55 in non-small cell lung cancer
Source: Mol Cancer. 2015 Apr 1;14:73. doi: 10.1186/s12943-015-0342-0 (PMC4392735; doi:10.1186/s12943-015-0342-0)
Supplement: Additional file 5: Table S1. — Correlations between GPR55 expression and clinicopathological variables in patients with NSCLC. [file 12943_2015_342_MOESM5_ESM.doc]

Additional file 5：Table S1 Correlations between GPR55 expression and clinicopathological variables in patients with NSCLC.

| **Table S1. Correlations between GPR55 expression and clinicopathological variables in patients with NSCLC** | | | | | | |
| --- | --- | --- | --- | --- | --- | --- |
| Variables | Total | GPR55 expression | | | X^2^ | *P*-value^a^ |
| Age(years)^b^ |  | + | ++ | +++ |  |  |
| ＜57 | 58 | 7 | 18 | 33 | 4.648 | 0.0986 |
| ≥57 | 22 | 7 | 4 | 11 |  |  |
|  |  |  |  |  |  |  |
| Gender |  |  |  |  |  |  |
| Male | 33 | 6 | 10 | 17 | 0.2994 | 0.8637 |
| Female | 47 | 8 | 12 | 27 |  |  |
|  |  |  |  |  |  |  |
| Smoking history(years)^b^ |  |  |  |  |  |  |
| ＜10 | 36 | 9 | 11 | 16 | 3.6521` | 0.1781 |
| ≥10 | 44 | 5 | 11 | 28 |  |  |
|  |  |  |  |  |  |  |
| Pathological type |  |  |  |  |  |  |
| adenocarcinoma | 38 | 8 | 12 | 18 | 1.7263 | 0.4391 |
| squamous carcinoma | 42 | 6 | 10 | 26 |  |  |
|  |  |  |  |  |  |  |
| Tumor differentiation |  |  |  |  |  |  |
| Ⅰ+Ⅱ | 46 | 9 | 16 | 21 | 4.0708 | 0.1439 |
| Ⅲ+Ⅳ | 34 | 5 | 6 | 23 |  |  |
|  |  |  |  |  |  |  |
| TNM Classification |  |  |  |  |  |  |
| Ⅰ | 35 | 10 | 13 | 12 | 11.5627 | 0.020 |
| Ⅱ | 27 | 2 | 5 | 20 |  |  |
| Ⅲ+Ⅳ | 18 | 2 | 4 | 12 |  |  |
|  |  |  |  |  |  |  |
| Metastasis |  |  |  |  |  |  |
| No | 44 | 9 | 17 | 18 | 8.4270 | 0.0112 |
| Yes | 36 | 5 | 5 | 26 |  |  |
| Abbreviations：TNM，tumor-node-metastasis;^a^x^2^test.^b^Mean age | | | | | | |
